# Influence of Thermal Environment on the Circulation and the Body-Heat

**Published:** 1913-09

**Authors:** 


**Influence of Thermal Environment on the Circulation and the Body-Heat.** By Edgar R. Lyth, M.B., M.R.C.S. Pp. iv, 72. London: John Bale, Sons & Danielsson Ltd. 1913. Price, 2s. 6d. net.

This interesting series of studies deals with the changes brought about in the state of the circulation and heat of the body by increasing or reducing the surrounding temperature. The experiments have been carried out very largely on the author himself, and the conclusions are said to be based upon more than twenty-five thousand observations of the pulse rate, the blood pressure and the superficial and deep temperatures of the body under various conditions.

On exposing the body to cold air, it is found that the pulse rate falls off, although, owing to the contraction of the peripheral vessels, the blood pressure rises. Passing into a hot atmosphere brings about reverse changes—the pulse rate increases, but the relaxation of the peripheral vessels induces a fall of blood pressure. Interesting observations on the changes produced by immersion of the body in hot and cold water are also set forth in considerable detail.

Perhaps the most remarkable chapter is that dealing with the influence of wind on the pulse rate, when some really striking figures are produced. To give a few examples. It is stated that an average pulse rate of 79.4 beats per minute inside a moving horse bus rises to 96 on going on the top, whilst similar figures in the case of a tramcar are 75.5 inside and 100 out. When the car stops, the number of beats falls until the normal 72 is reached, but as soon as the car starts again the pulse rate goes up to its previous figure. Continued exposure to wind may raise the number of pulse beats to as many as 150 per minute. That this stimulating effect is due to the wind is supported by the fact that in a sheltered position inside a railway train moving at express speed the pulse remains quite normal. A somewhat similar action on the pulse rate is noted when the body is immersed in water, and the water set in motion by gently stirring.

The general conclusion arrived at by the author is that in a given thermal environment a certain corresponding equilibrium of the circulatory apparatus ultimately becomes established, comprising, as regards the blood-vessels, a suitable degree of muscular contraction of their coats, and as regards the heart a suitable rate and force of its pulsations. Besides determining the pressure and the speed of the current, these factors involve a corresponding distribution of the blood in the skin and in the arterial vessels.

The results of the various kinds of experiments are well illustrated by diagrams and lucidly discussed, and will well repay careful consideration from the medical standpoint.

